# IER3: exploring its dual function as an oncogene and tumor suppressor

**DOI:** 10.1038/s41417-025-00891-y

**Published:** 2025-03-16

**Authors:** Meena Kanduri, Santhilal Subhash, Rossana Putino, Sagar Mahale, Chandrasekhar Kanduri

**Affiliations:** 1 Department of Laboratory Medicine, Institute of Biomedicine, Sahlgrenska Academy, University of Gothenburg, Gothenburg, Sweden; 2 Department of Medical Biochemistry and Cell Biology, Institute of Biomedicine, Sahlgrenska Academy, University of Gothenburg, Gothenburg, Sweden; 3 Cold Spring Harbor Laboratory, Cold Spring Harbor, NY USA; 4 Department of Biosciences and Bioengineering, Indian Institute of Technology Jammu, Jammu, India

**Keywords:** Oncogenes, Cancer genetics

## Abstract

The IER3 gene has a complex role in cancer biology, acting either as a tumor suppressor or an oncogene, depending on the cancer type. This duality underscores the complexity and importance of molecular pathways in modulating cancer behavior. Despite its significance in cancer development, there is a dearth of studies elucidating the exact mechanisms underlying IER3’s involvement in modulating cancer behavior. Here, utilizing cervical carcinoma and neuroblastoma (NB) cell lines as model systems we characterized the pathways that mediate the functional switch between the oncogenic and tumor suppressor roles of IER3. In HeLa cells, IER3 expression promotes an oncogenic program that includes immediate early response pathway genes such as EGR2, FOS, and JUN. However, in NB cells, IER3 suppresses the EGR2-dependent oncogenic program. This differential regulation of EGR2 by IER3 involves epigenetic modulation of the EGR2 promoter. IER3 dependent tumor suppressor pathway in NB cells relies on ADAM19 gene. Thus, our findings uncover the molecular pathways that dictate the context-dependent roles of IER3 in cancer, providing insights into its dual functionality in different cancer types.

## Introduction

Among the numerous genes that play pivotal roles in cellular responses to external stimuli, the Immediate Early Response 3 (IER3) gene has emerged as a key player. Also known as IEX-1, this gene has garnered increasing attention due to its multifaceted functions across various biological contexts. It belongs to the family of immediate early response genes, which includes well-characterized members such as c-fos, c-jun, and c-myc [1, 2]. IER3 is a stress-inducible gene and exerts divergent effects on cellular responses under stress conditions [3, 4], playing a pivotal role in regulating cell apoptosis, proliferation, differentiation, and DNA repair [5].

IER3 can be rapidly and transiently induced in response to a wide array of stimuli, including growth factors, cytokines, ionizing radiation, viral infections, and other forms of cellular stress [6]. Its effects on cell survival, apoptosis, proliferation, and differentiation vary depending on the cancer cell type, either inhibiting or facilitating cancer progression [6, 7]. Consequently, IER3 has a complex and often paradoxical role in regulating the cell cycle and apoptosis [8], rendering a survival advantage to certain cancer types by blocking cell differentiation, while promoting apoptosis in others through mechanisms that are not yet fully understood [1, 2, 9].

At the molecular level, IER3 is deeply involved in regulating critical signaling pathways, including NF-κB, MAPK/ERK, and PI3K/Akt [1, 2, 10]. IER3 affects cell differentiation and proliferation by regulating the MAPK/ERK signaling pathways and modulating ubiquitin-proteasome activity [11, 12]. Additionally, under stress conditions that cause DNA damage, IER3 can either promote DNA repair or induce apoptosis by suppressing NF-κB transcriptional activity in the nucleus [10]. Reflecting its complex role, differential IER3 expression has been observed in various human cancers, correlating with either poor or favorable prognosis depending on the cancer type and stage of progression. This makes IER3 a potentially valuable biomarker for cancer diagnosis and prognosis [2, 13–15].

Despite extensive studies on IER3 in cancer, there is still a lack of research clarifying how this gene switches between oncogenic and tumor suppressor functions depending on the cellular context and specific stimuli. In line with this complexity, we recently demonstrated oncogenic functions of IER3 in cervical (HeLa) and breast (BT-549) cancer cells. Our findings revealed that the oncogenic properties of IER3 depend on interactions with the RNA-binding protein HnRNPK and molecular cross-talk with its antisense RNA partner, IER3-AS1 [16]. When we investigated cancers where IER3 displays tumor suppressor functions, we identified neuroblastoma (NB) tumors in which higher IER3 expression correlates with good prognosis. Thus, cell lines representing cervical carcinoma and NB would be ideal model systems to address the oncogenic and tumor suppressor functions of IER3, respectively.

NB is an interesting childhood cancer type that predominantly occurs in the adrenal gland. It is a tumor of peripheral nervous system, thought to arise due to disturbances in the differentiation of neural crest cells into mature neurons of peripheral nervous system, thereby giving rise to several intermediary cell types, including neuroblasts [17–20]. These tumors account for almost 15% of all pediatric cancer deaths [20, 21]. One unique feature associated with NB is that a significant number of low-risk patients undergo spontaneous tumor regression [22], and the underlying mechanisms to this very interesting phenomenon still remain unknown. Thus, understanding IER3-dependent tumor suppressor functions could provide significant insights into the molecular basis of this regression and potentially inform new therapeutic strategies.

In this study, we explored the dual role of IER3 as both an oncogene and a tumor suppressor. Our study characterizes the pathways that mediate the functional switch between the oncogenic and tumor suppressor roles of IER3. Notably, we demonstrate that the oncogenic functions of IER3, such as increased cell proliferation, inhibition of apoptosis, and S-phase prolongation due to cell cycle arrest, are mediated through the EGR2 oncogene by activating the downstream immediate early response pathway comprising JUN and FOS genes. Conversely, the tumor suppressor functions, such as inhibition of cell invasion and migration, are primarily regulated through the ADAM19 gene. These findings underscore the IER3-dependent differential expression of target genes between HeLa and NB cell lines, correlating with its distinct functions in these two tumor types.

## Results

### IER3 is a P53 responsive gene in NB

Previously, we characterized the p53-regulated gene expression profile after treating NB cell lines with Nutlin and Selinexor to activate p53 [23]. In that study, we identified IER3 as one of the top genes with increased expression upon p53 induction. To further confirm whether IER3 is a p53-responsive gene in NB cell lines, we conducted transient transfections using p53 siRNA in two NB cell lines, SH-SY5Y and SK-N-BE(2), and assessed IER3 expression. The data indicate a significant reduction in IER3 mRNA and protein levels upon p53 knockdown in the SH-SY5Y cell line, which contains wild-type p53. However, in the SK-N-BE(2) cell line, where p53 is mutated, IER3 expression showed no significant change. (Fig. S1A, B). These data confirms that IER3 is a p53 responsive gene in NB.

### IER3 acts as a tumor suppressive gene in NB

**Fig. 1:**
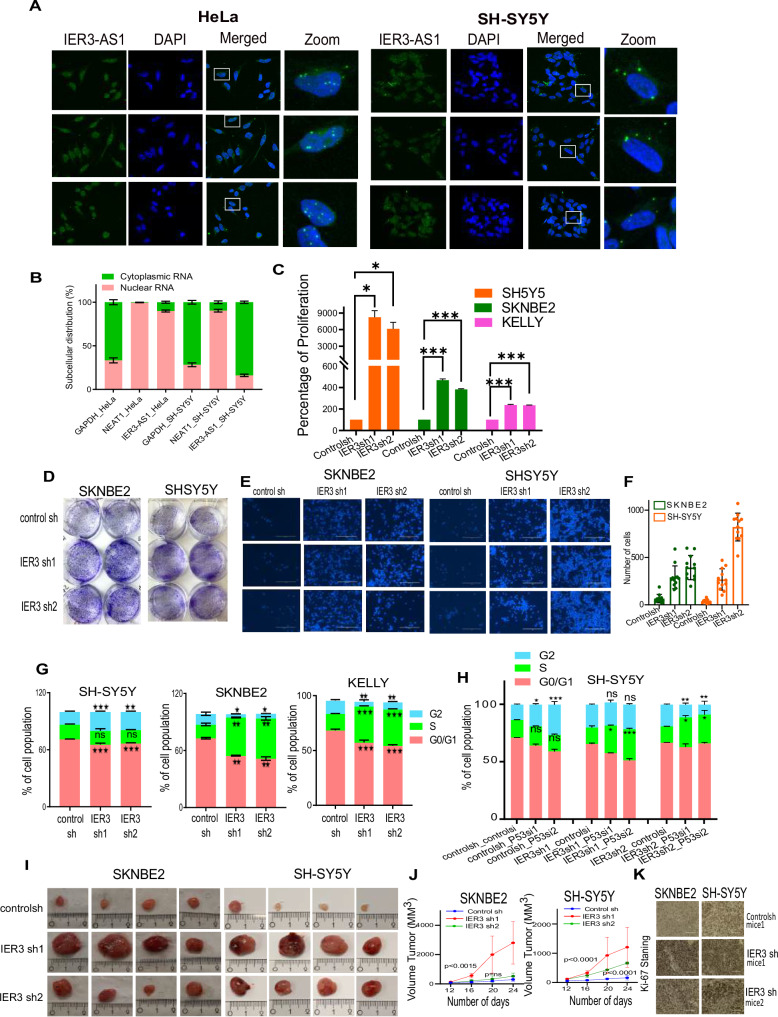
IER3 has Tumor suppressor properties in Neuroblastoma. **A** RNA Scope images showing IER3-AS1 transcripts in HeLa and SH-SY5Y cells. Images at the right represents the magnified areas of the indicated white boxes shown in Merged images. DAPI was used to stain nucleus (Indicative scale bar is 50 µm). **B** RT–qPCR data showing the nuclear and cytoplasmic distribution of IER3-AS1. GAPDH and NEAT1in HeLa and SH-SY5Y cells. **C** Cell Viability Assay showing the percentage of cell proliferation in IER3 downregulated (KD) NB cell lines. **D** Colony forming efficiency of IER3 downregulated (KD) SH-SY5Y and SK-N-BE(2) cell lines. **E** Transwell Invasion assay showing the invasive potential of IER3 in SH-SY5Y and SK-N-BE(2) KO stable cell lines. **F** Bar graphs shows quantification of the invasive cells shown in Figure G. The invasive cells were counted using ImageJ software. **G**, **H** Distribution plots showing the percentage of cell populations of IER3 stable KD SH-SY5Y, SK-N-BE(2) and KELLY cells in G0/G1, S and G2 phases of cell cycle. **I** Representative images of tumors (*n* = 4) depicting the tumor size of xenografts that were developed by subcutaneously injected SH-SY5Y and SK-N-BE(2) cells stably transduced with control sh or *IER3* shRNA. Tumors were harvested after 25 days post injection. **J** The graph showing the difference in the volume of tumors between NB control sh mice and NB IER3sh mice group (each group contains 4 mice, Random selection). **K** Representative images of the Ki67 staining on the xenografts derived from mice tumor cross sections. The data shown here is from three individual mice tumors. All values represent mean ± SD of three biological experiments. ∗*p* < 0.05, ∗∗*p* < 0.01 and ∗∗∗*p* < 0.001 by Student’s *t* test and two-way ANNOVA.

In our recent publication, we demonstrated that IER3 exhibits oncogenic properties in cervical carcinoma, lung adenocarcinoma, and breast cancer cell lines [16]. However, our observations on clinical data from different NB tumor cohorts revealed that IER3 harbors tumor suppressor properties. Higher expression of IER3 in NB patients predicts a favorable prognosis, while its low expression associated with worse prognosis. Additionally, IER3 and MYCN exhibit an inverse relationship in expression among neuroblastoma (NB) patients, where low IER3 levels combined with high MYCN levels are associated with poor survival in the same patient cohort (R2 Genomics Platform) (Fig. S1E). To address the role of IER3 in NB, we generated IER3 loss-of-function (shRNA) stable cell lines in three NB cell lines (SH-SY5Y, SK-N-BE(2) and KELLY) (Fig. S1C, D). Previously, we demonstrated that IER3 and its antisense RNA counterpart, IER3-AS1, regulate each other’s expression and contribute to oncogenic properties in cervical, lung, and breast carcinoma cell lines [16]. Given that IER3 display tumor suppressor properties in NB, we explored the impact of IER3 downregulation on IER3-AS1 expression, and vice versa, in NB cell lines. Neither IER3 nor IER3-AS1 downregulation affected the expression of the other gene (Fig. S2A). To understand IER3-dependent tumor suppressor mechanisms in NB, we performed RNA sequencing of two IER3KD NB stable cell lines (one p53 wild-type SH-SY5Y; and one p53 mutated SK-N-BE(2). The total list of significant differentially expressed DEGs from controls vs IER3 sh KD samples for three NB cell lines are listed in Supplementary datafile 1. Expression levels of IER3 and IER3-AS1 in RNA-seq analysis of stably KD IER3 cell lines also confirms that IER3 does not regulate the expression of IER3-AS1 (Fig. S2B). Notably, RNA in situ hybridization and subcellular RNA distribution assays revealed that IER3-AS1 transcripts were primarily nuclear in HeLa cells, whereas in SH-SY5Y cells, IER3-AS1 transcripts were predominantly localized in the cytoplasm (Fig. 1A, B). The specificity of the IER3-AS1 probe was tested using IER3-AS1 shRNA and RNase A treatment in HeLa cells, as demonstrated in our previous study [16]. Additionally, the expression correlation between IER3 and IER3-AS1 transcripts was weaker in NB cohorts from the R2 database, in contrast to the breast, cervical, and lung cancer cohorts, which exhibited a significant positive correlation between these two genes (Fig. S2C). Taken together, these data suggest that IER3-AS1 and IER3 functions are not interdependent, and that IER3-AS1 operates independently of IER3 in NB cells.

To determine if IER3 has any tumor suppressor role in NB, we performed several functional assays that explore the crucial cancer cell hallmarks such as cell proliferation assay, colonization assay, cell invasion assay and cell cycle analysis using the stably maintained shRNA KD IER3 NB cell lines. These assays revealed a significant increase in cell proliferation, cell colonization and cell invasion upon IER3 KD in NB cell lines (Fig. 1C–F). Additionally, cell cycle analysis revealed that IER3 KD exerts cell cycle arrest in the S-Phase, resulting in a significant increase and prolonged S-phase (Fig. 1G). This effect was more pronounced in SK-N-BE(2) and KELLY cell lines, where the tumor suppressor protein p53 is known to be mutated, compared to SH-SY5Y cells, which harbors wild-type p53 (Fig. 1G). Upon down-regulation of p53 in the SH-SY5Y IER3KD cell line, a significant increase in S-phase was observed, mirroring the phenotype seen in SK-N-BE(2) and KELLY (Fig. 1H). These findings collectively suggest that IER3 plays a cooperative role with p53 in regulating S-phase cell cycle arrest in NB. Furthermore, the subcutaneous injections of SH-SY5Y and SK-N-BE(2) cell lines lacking IER3 (IER3sh cell lines) in NSG mice showed a significant increase in tumor weight and tumor size, indicating that loss of IER3 results in increased tumor growth (Fig. 1I–K). All the above-mentioned functional data clearly indicate that IER3 possess putative tumor suppressor properties in NB cells.

### Characterization of IER3 mediated Pro-survival pathways

**Fig. 2:**
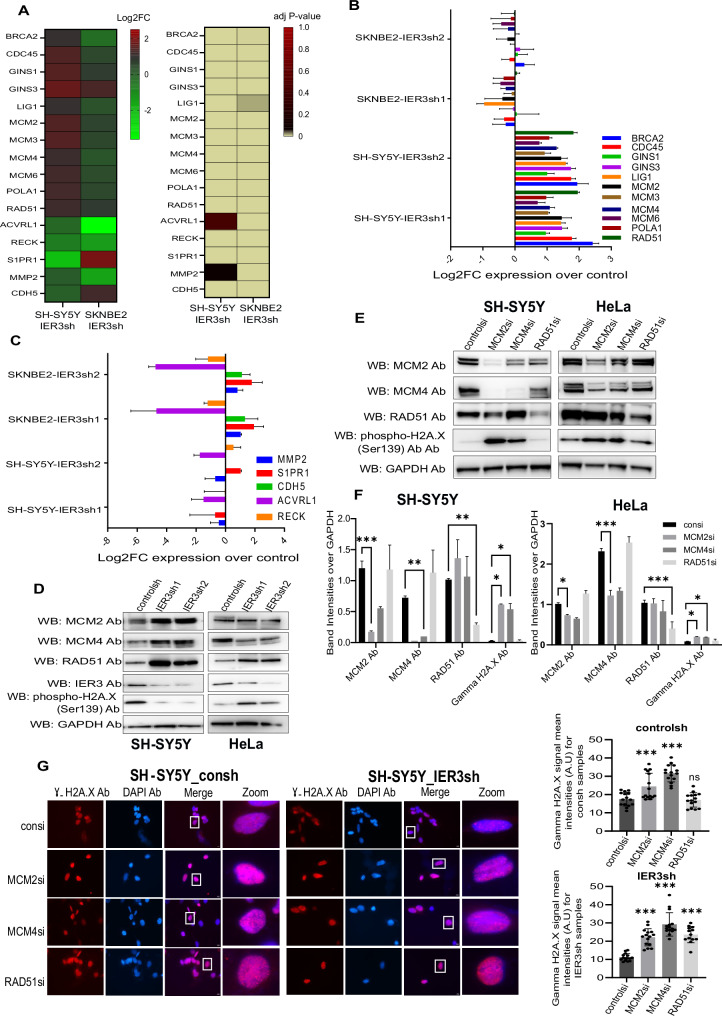
Characterization of IER3 mediated pathways and responsible genes in different NB cell lines. **A** Heatmaps showing the log2FC values of IER3 target genes that were significantly upregulated and downregulated in DNA replication and Blood vessel maturation pathways in both SH-SY5Y and SK-N-BE(2) cell lines. The bar showing the color codes (on the right side) indicates Log2FC and p-value respectively for both the Heatmaps. **B**, **C** Validation using RT-qPCR showing the levels of IER3 mediated pathway genes in both NB cell lines. Log2FC expression values of IER3sh were presented over controlsh samples. **D**, **E** Western blot showing the protein expression levels of IER3 and its target genes in SH-SY5Y and HeLa cells. **F** Quantitation of Western blots shown in (**E**). Bars show the mean band intensity from two independent Western blots, normalized to GAPDH band intensities. Error bars show the standard error of the mean intensities and statistical significance was calculated by Two-way ANOVA. *p < 0.05. **p < 0.001 ***p < 0.0001. **G** Immunostaining images showing Phospho gammaH2A.X antibody (Red) in control and IER3sh KD SH-SY5Y cells. Images at the right (zoom) represents the magnified areas of the indicated white boxes from the merged images. DAPI was used to stain nucleus (Indicative scale bar is 50 µm). The Graph in the right side shows the numbers of RNA signals (spots) per cell in control sh and IER3 sh samples counted using ImageJ. Total number of cells counted from three independent replicates are 45 cells for each sample. Data represent mean values ± SEM and statistical significance was calculated by Two-way ANOVA.

The functional enrichment/GO pathway analysis of RNA-seq data from IER3 KD cells revealed pathways specific to SH-SY5Y (DNA replication and initiation pathways), and to SK-N-BE(2) and KELLY (for example blood vessel maturation pathway) (Fig. S3A, B and Fig. 2A). The top genes responsible for these pathways were selected from RNA seq data for both SH-SY5Y and SK-N-BE(2) cell lines (shown in Fig. 2A) are validated using qRT-PCR analysis (Fig. 2B, C).

Interestingly, all the top 25 GO pathways for SH-SY5Y IER3KD were related to the initiation of DNA replication pathway (Fig. S3A). To further confirm the role of IER3 in DNA replication within SH-SY5Y cells, we selected three DNA replication genes—two mini-chromosome maintenance proteins (MCM2 and MCM4), involved in the initiation of eukaryotic genome replication [24], and RAD51, known for its role in DNA repair [25]. These genes exhibited high expression levels exclusively in the SH-SY5Y cell line, compared to the SK-N-BE(2) and HeLa cell lines, according to IER3sh KD RNA sequencing data. The protein expression levels for these three genes were validated in SH-SY5Y and HeLa IER3sh cell lines (Fig. 2D).

Furthermore, siRNA-mediated downregulation of these genes in SH-SY5Y control and IER3sh KD cell lines resulted in an increase in DNA double-stranded breaks, as analyzed using γH2AX immunoblotting (Fig. 2E, F). However, fewer γH2AX foci were observed in SH-SY5Y IER3sh KD cells, which had higher expression levels of these genes compared to SH-SY5Y control cells (Fig. 2G). These findings confirm the involvement of IER3 in DNA replication and repair functions in SH-SY5Y cells.

### Characterization of IER3 dependent oncogenic and tumor suppressor pathways

**Fig. 3:**
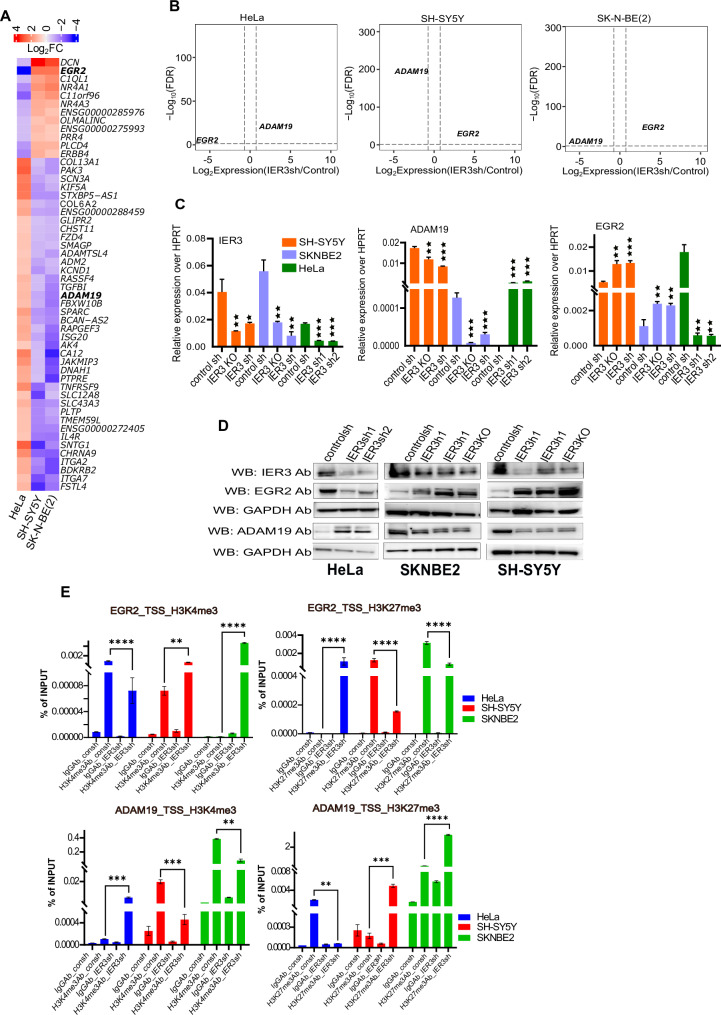
Comparing the oncogenic and Tumor suppressor functions of IER3 in both HeLa and NB cell lines. **A** Heatmap showing the expression (log2FC values) from RNA sequencing data of consh vs IER3sh DEGs Hela cells compared to NB cell lines. The bar on the above represents the color code for Log2FC values. **B** Volcano plots showing Log10FDR on Y axis and LogFC on X-axis of DEGs from RNA-seq data of HeLa and NB cell lines. Up and down regulated DEGs are shown in red and blue color dots respectively. The non-significant DEGs are represented as gray dots. FDR threshold (=0.05) is reported as horizontal dotted gray line. FDR values are derived from DESeq2 R-package by adjusting p-values using Benjamini-Hochbergmethod. **C** RT-qPCR validations of the gene expression levels of IER3, ADAM19 and EGR2 in Hela and NB cell lines using consh and IER3sh samples. **D** Western blot analysis showing the protein levels for all the different mentioned antibodies using consh and IER3sh samples in HeLa and NB cell lines. **E** Real time-qPCR ChIP assay data showing percentage of Input for histone active promoter mark (H3K4me3) and inactive promoter mark (H3k27me3) on IER3 target gene promoters (EGR2 and ADAM19) in HeLa and NB cell lines. All values represent mean ± SD of two to three biological experiments. ∗*p* < 0.05, ∗∗*p* < 0.01 and ∗∗∗*p* < 0.001 by Student’s *t* test.

To further delineate the functions of IER3 in cervical and NB cancer models, we analyzed common genes that are differentially regulated in these two cancer models (HeLa vs. SH-SY5Y and SK-N-BE(2)) (Fig. 3A). The functional enrichment and GO pathway analysis for these comparisons are listed in Supplementary Datafile 1. Among the genes that exhibited significant differential expression between HeLa IER3 KD and SH-SY5Y/SK-N-BE(2) IER3 KD comparisons, EGR2 and ADAM19 were selected for further analysis due to their diverse roles in cancer, functioning as both tumor suppressors and oncogenes depending on the cell type, similar to IER3.

EGR2 and ADAM19 exhibit opposing expression patterns in HeLa and NB cell lines upon IER3 downregulation (Fig. 3B). We further validated the RNA-seq data through RT-qPCR and Western blot analyses in all three cell lines—HeLa, SH-SY5Y, and SK-N-BE(2)—with IER3 downregulated using either shRNA or CRISPR knockout (CRISPR promoter knockout) (Fig. 3C, D). EGR2 expression was found to be high in NB cell lines and low in HeLa cell lines upon IER3 downregulation, consistent with oncogenic properties of EGR2. Conversely, ADAM19, known for its tumor suppressor properties, exhibited higher expression in HeLa and lower expression in NB upon IER3 (shRNA) downregulation (Fig. 3C, D).

Given that both EGR2 and ADAM19 show differential expression patterns in HeLa and NB cell lines, we investigated chromatin organization at their promoter regions by performing ChIP assays using antibodies against the active chromatin mark H3K4me3 and the inactive chromatin mark H3K27me3 on control and IER3 KD cells. The results showed a significant decrease in H3K4me3 and an increase in H3K27me3 occupancy at the EGR2 promoter in IER3 KD HeLa cells compared to control HeLa cells. Conversely, in both SH-SY5Y and SK-N-BE(2) IER3 KD cells, we observed the opposite pattern (Fig. 3E). At the ADAM19 promoter, HeLa IER3 KD cells showed increased H3K4me3 and reduced H3K27me3 levels compared to control HeLa cells. In contrast, NB cell lines exhibited the opposite pattern, aligning with their differential expression profiles (Fig. 3E).

### Analysis of IER3 occupancy on its target genes

**Fig. 4:**
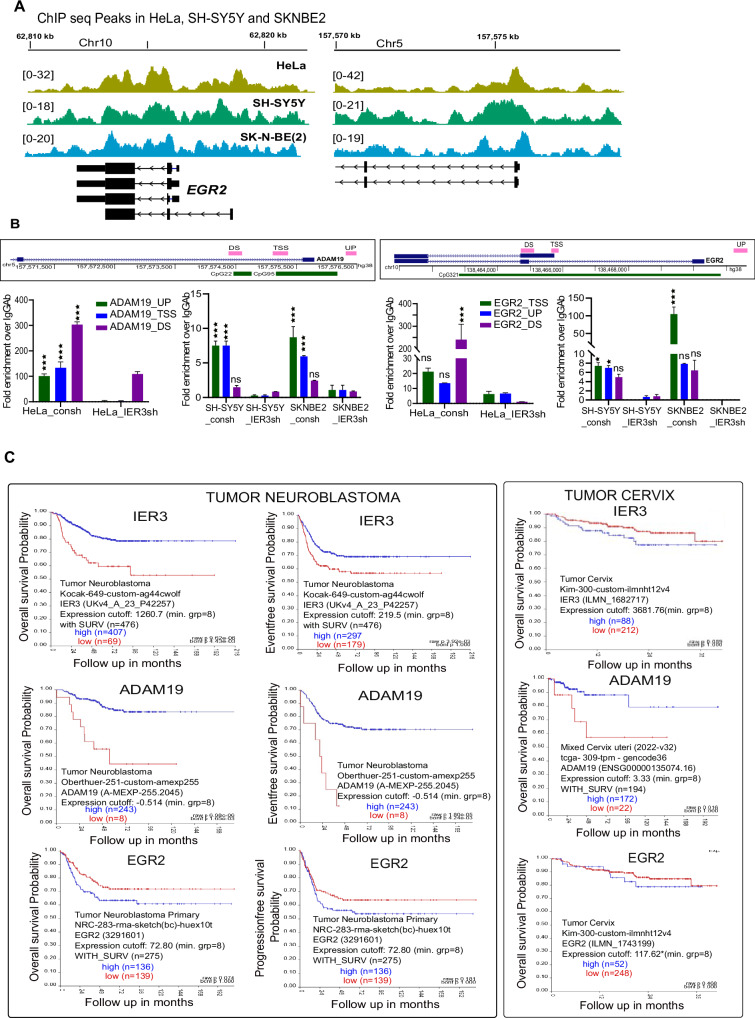
Binding of IER3 on its target genes and survival probability of IER3, EGR2 and ADAM19 in both tumor types. **A** Screenshot of the IGV Genome Browser comparing the peaks from IER3 ChIP sequencing data on EGR2 and ADAM19 genes. The IER3 pull down peaks are normalized against respective ChIP input peaks. The data shown here is for HeLa and NB cell lines. **B** Schematic representation of the ChIP primer locations on the EGR2 and ADAM19 promoter regions. Below is the RT-qPCR ChIP assay data showing Fold enrichment of IER3 over IgG on EGR2 and ADAM19 promoters in HeLa and NB cell lines. **C** Kaplan–Meier curves for IER3, EGR2 and ADAM19 genes showing both overall survival probability and Eventfree survival probability in both Neuroblastoma and Tumor cervix data sets. The *p* value and the tumor sample numbers used in the analysis are mentioned on the plots.

Considering the dual role of IER3 as both an oncogene and a tumor suppressor, we sought to explore IER3 protein occupancy on a genome-wide scale. To achieve this, we performed ChIP sequencing using an IER3 antibody in NB and HeLa cell lines, and compared the ChIP-seq data between these cell types (HeLa vs. SH-SY5Y and HeLa vs. SK-N-BE(2)). Notably, both EGR2 and ADAM19 showed significant IER3 ChIP-seq peaks in their promoter regions, indicating IER3 occupancy at these gene promoters (Fig. 4A). Additionally, we validated the ChIP-seq data through ChIP-qPCR assays in both NB and HeLa cell lines for the EGR2 and ADAM19 genes (Fig. 4B). These results confirm that IER3 binds to the promoters of these two target genes, thereby regulating its functions in both HeLa and NB cell lines.

Subsequently, we examined the clinical implications of EGR2 and ADAM19 expression in NB and cervical carcinoma patient cohorts using a published database (R2 visualization database). Interestingly, higher expression of EGR2 predicted poor overall survival and progression-free survival in NB and cervical carcinoma patients (Fig. 4C). Given the observed increase in EGR2 expression in NB cell lines and decrease in HeLa cells following IER3 downregulation, the clinical data aligns with its oncogenic functions in these cancers. Similarly, high expression of ADAM19 predicted a favorable prognosis in both NB and cervical carcinoma patients, consistent with its differential expression following IER3 downregulation (Fig. 4C). In HeLa cells, where ADAM19 expression is increased following IER3 downregulation, it correlates with a decrease in tumor size, while in NB cell lines, where ADAM19 expression is decreased upon the IER3 downregulation, it correlates with an increase in tumor size.

In conclusion, our data strongly suggests that both EGR2 and ADAM19 genes are bona fide targets of IER3, and that IER3 executes its functions as a tumor suppressor or oncogene by differentially controlling the expression of these genes.

### IER3 mediates its oncogenic functions through the EGR2-regulated C-JUN and FOS pathway

**Fig. 5:**
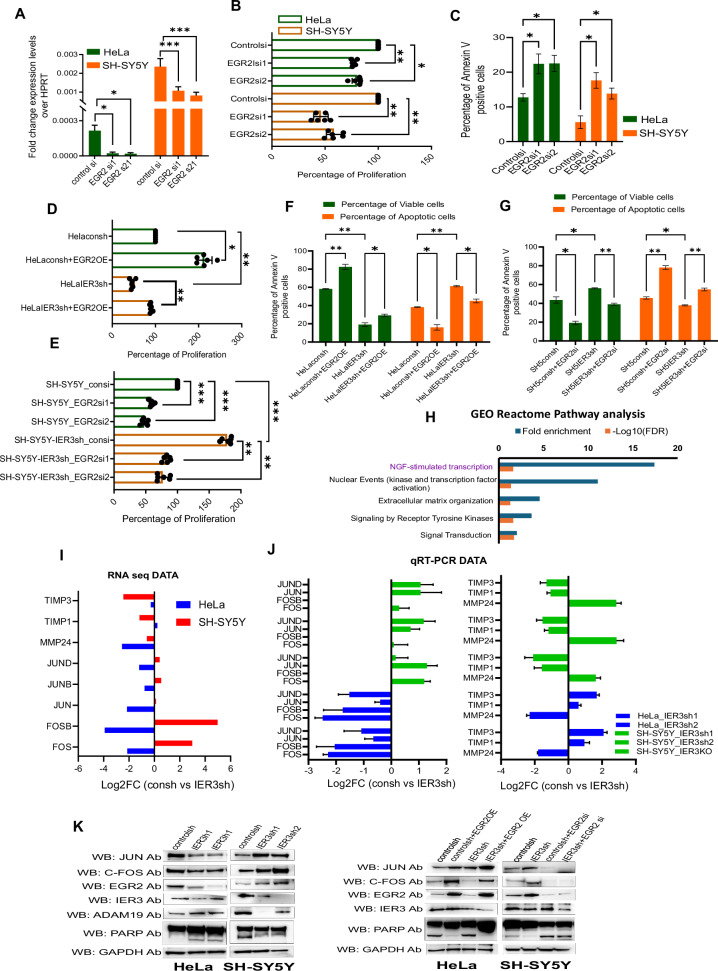
IER3 exerts oncogenic functions through EGR2 by activating C-JUN/FOS pathway. **A** RT-qPCR expression levels of EGR2 in EGR2 siRNA KD samples of HeLa and SH-SY5Y cells. **B** Cell Viability Assay showing the percentage of cell proliferation in both EGR2 siRNA downregulated SH-SY5Y and SK-N-BE(2) cell lines. **C** Bar graphs showing percentage of Annexin V positive cells in EGR2 siRNA down regulated samples in NB cells. **D**–**G** Cell Viability Assay showing the percentage of cell proliferation in EGR2 OE in HeLa cells and EGR2 siRNA downregulated samples in NB cell lines. **H** Functional gene enrichment analysis for differentially expressed HeLa vs SH-SY5Y consh vs IER3sh DEGs. **I** Bar graph representing the Log2FC levels of C-JUN, FOSB pathway genes from RNA sequencing data. **J** RT-qPCR data validating the Log2FC levels of consh vs IER3sh expression levels of C-JUN, FOSB pathway genes in HeLa and SH-SY5Y cells. **K** Western blot analysis showing the protein levels for all the different mentioned antibodies using consh and IER3sh samples in HeLa and NB cell lines. All values represent mean ± SD of three biological experiments. ∗*p* < 0.05, ∗∗*p* < 0.01 and ∗∗∗*p* < 0.001 by Student’s *t* test.

Our RNA-seq and ChIP-seq data clearly indicate that EGR2 is a bona fide target of IER3, playing a crucial role in mediating IER3-dependent oncogenic functions. Additionally, clinical data further support the oncogenic activity of EGR2. To further validate its role, we downregulated EGR2 in HeLa and NB cell lines to determine whether it could replicate the cancer cell hallmark phenotypes observed with IER3 downregulation. Functional assays revealed that siRNA-mediated EGR2 downregulation in both HeLa and NB cell lines led to decreased cell proliferation and increased apoptosis (Fig. 5A–C).

To investigate whether EGR2 can reverse the effects of IER3 knockdown (KD), we downregulated EGR2 in IER3 KD SH-SY5Y cells and overexpressed EGR2 in IER3 KD HeLa cells. Our cell proliferation and apoptosis assays revealed that EGR2 overexpression in HeLa cells led to increased cell proliferation and reduced apoptosis, while EGR2 downregulation in SH-SY5Y cells had the opposite effect (Fig. 5D–G). These results indicate that EGR2 functions downstream of IER3 and can rescue the phenotypes associated with IER3 KD in these cell types.

Considering the role of EGR2 in IER3-dependent oncogenic functions, we sought to identify the downstream gene network through which EGR2 exerts its oncogenic effects. GO analysis of IER3 KD HeLa and SH-SY5Y cells revealed that NGF-stimulated transcription and signaling by receptor tyrosine kinase pathways were among the top pathways identified (Fig. 5H). Nerve growth factor (NGF) is known to activate the FosB/Jun/AP-1 pathway, which plays a crucial role in cellular processes such as growth, differentiation, survival, and response to external stimuli, particularly in neurons [26].

EGR2 activates the c-Jun/Fos pathway either directly, or indirectly through downstream signaling pathways such as MAPK and PI3K/AKT [27–29]. The induction of the c-Jun/Fos pathway by EGR2 can contribute to cancer progression by promoting cell proliferation and survival in various cancers [30]. We found that both C-JUN and FOS genes were significantly differentially expressed in our RNA-seq data from IER3 knockdown (KD) cells, along with several downstream target genes, including MMP24, as well as two negative regulator genes, TIMP1 and TIMP3 (Fig. 5I; Fig. S4D). The expression levels of all these genes were validated using qRT-PCR in both HeLa and SH-SY5Y IER3 KD cell lines (Fig. 5J).

Subsequently, we examined the role of EGR2 in the IER3-dependent differential expression of the JUN and FOS genes. To address this, we utilized IER3 KD HeLa and NB cell lines, overexpressing EGR2 in IER KD HeLa cells, while downregulating EGR2 in IER3 KD SH-SY5Y cells. We found that IER3 KD in HeLa cells resulted in reduced expression of the JUN and FOS genes, whereas their expression increased upon IER3 downregulation in SH-SY5Y cells. The reduced expression of JUN and FOS in HeLa cells following IER3 KD is consistent with the decreased expression of EGR2, highlighting the critical role of the IER3-EGR2 pathway in regulating the downstream FOS/JUN network. Additionally, the diminished EGR2-FOS/JUN pathway in IER3 KD HeLa cells correlated with increased levels of the apoptotic markers, such as cleaved PARP (Fig. 5K).

To further investigate the role of EGR2 in IER3-dependent FOS/JUN expression, we overexpressed EGR2 in IER3 KD HeLa cells and downregulated EGR2 in IER3 KD SH-SY5Y cells. Our results showed that EGR2 overexpression restored the expression of the FOS/JUN genes in IER3 KD HeLa cells, which was associated with decreased levels of the apoptotic markers. Conversely, downregulating EGR2 in IER3 KD SH-SY5Y cells resulted in reduced expression of the FOS/JUN genes, correlating with an increase in the apoptotic markers (Fig. 5K). Thus, these results confirm that the IER3-dependent oncogenic functions are predominantly regulated through the EGR2-controlled FOS/JUN pathway (Fig. 5F, G, K and Fig. S4A–C).

### The tumor suppressor functions of IER3 are mediated through ADAM19

**Fig. 6:**
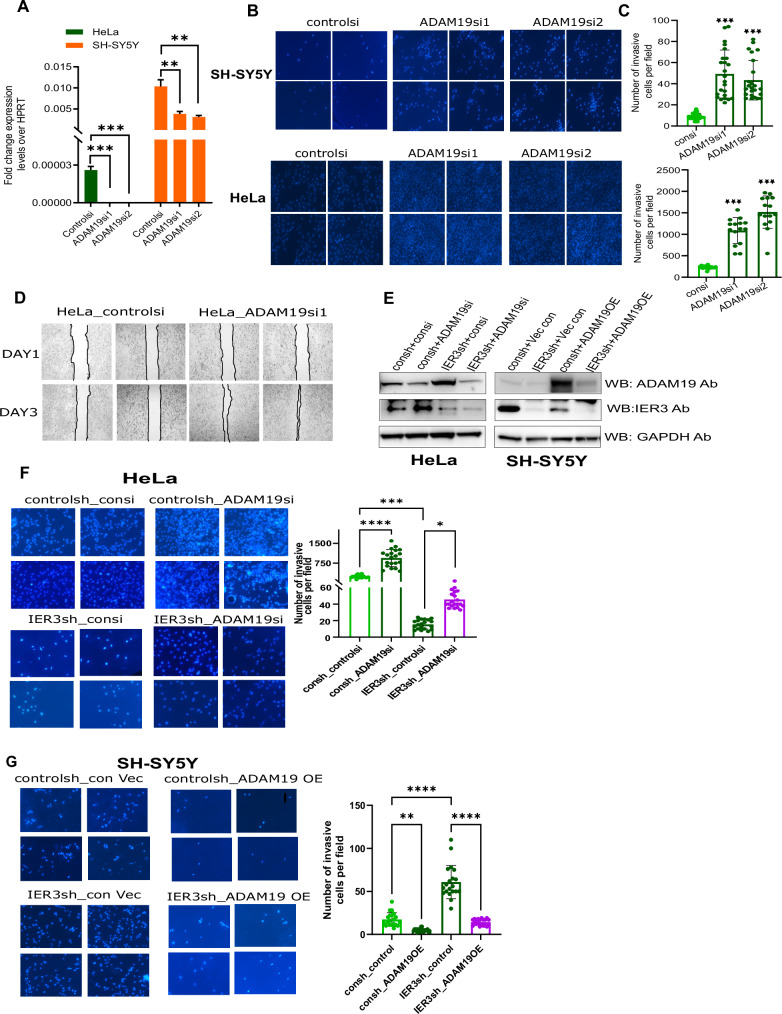
IER3 exerts Tumor suppressor functions through ADAM19 by suppressing cell migration and invasion in HeLa and NB cell lines. **A** RT-qPCR expression levels of ADAM19 in ADAM19 siRNA KO samples of HeLa and SH-SY5Y cells. **B** Transwell Invasion assay showing the invasive potential of ADAM19 in SH-SY5Y and SK-N-BE(2) KO stable cell lines. **C** Bar graphs shows quantification of the invasive cells shown in Figure B. The invasive cells were counted using ImageJ software. **D** Wound healing assay showing the cell migration capacity in HeLa cells comparing between controlsi and ADAM19siRNA transfected cells. **E** Western blot analysis showing the protein levels ADAM19 and IER3 ADAM19siRNA KD and ADAM19 overexpressed samples in HeLa and SH-SY5Y5 cells respectively. **F** Transwell Invasion assay showing the invasive potential of ADAM19 in HeLaconsh and HeLa IER3sh stable cell lines. Bar graphs on right side shows quantification of the invasive cells shown in Fig. 5F. The invasive cells were counted using ImageJ software. **G** Transwell invasion assay and Bar graph with quantification of ADAM19 invasive potential for ADAM19 overexpression samples in SH-SY5Y5consh and IER3sh stable cell lines. All values represent mean ± SD of three biological experiments. ∗*p* < 0.05, ∗∗*p* < 0.01 and ∗∗∗*p* < 0.001 by Student’s *t* test and Two-way ANOVA.

ADAM19 as a tumor suppressor has been shown to inhibit cancer cell proliferation, migration, and invasion by modulating the key pathways involved in cell growth and survival. Its activation in IER3 knockdown (KD) HeLa cells and downregulation in IER3 KD SH-SY5Y cells align with ADAM19’s tumor-suppressive role. We therefore explored the significance of ADAM19’s differential expression in HeLa and SH-SY5Y cells following IER3 knockdown. Although siRNA-mediated downregulation of ADAM19 in HeLa and SH-SY5Y cells, as shown in Fig. 6A, had no significant effect on cell proliferation or apoptosis (Fig. S4E, F), it notably increased cell migration and invasion in both HeLa and SH-SY5Y cells (Fig. 6B–D).

Next, to investigate whether ADAM19 could counteract the effects of IER3 knockdown, we overexpressed ADAM19 in IER3 KD SH-SY5Y cells and downregulated it in IER3 KD HeLa cells. Downregulation of ADAM19 in IER3 KD HeLa cells resulted in increased cell invasion, while its overexpression suppressed the invasive capacity of IER3 KD SH-SY5Y cells (Fig. 6E–G). These findings clearly demonstrate that ADAM19 plays a critical role in regulating both the oncogenic and tumor suppressor functions of IER3.

Overall, our data demonstrate that IER3 exhibits a dual role in cancer, acting as both an oncogene and a tumor suppressor, depending on the context. IER3 executes this dual role through its direct, cancer cell-specific effects on the EGR2/FOS/JUN and ADAM19 pathways. Thus, our work uncovers the molecular pathways that govern the context-dependent functionality of IER3 in cancer.

## Discussion

Immediate early genes are a group of genes rapidly activated by various cellular stimuli, such as growth factors and stress signals. Many members of this gene family are transcription factors that can quickly initiate gene transcription, while others are essential for enabling cells to respond promptly to stressors [1, 5]. Recent advances in the study of immediate early genes suggest they may play a significant role in regulating oncogenic responses. However, the expression and function of immediate early genes in cancer development and progression remain largely unexplored. Hence, further research is needed to understand the mechanisms by which immediate early genes influence cancer pathways and how they can be leveraged for therapeutic interventions.

A growing body of evidence suggests that IER3, an immediate early response gene, is associated with the prognosis of various cancers showing contradictory roles by enhancing apoptosis in certain cancers and promoting proliferation in others [5]. This makes IER3 a valuable gene for studying molecular pathways, potentially paving the way for innovative therapies. The direct role of IER3 in regulating the NF-kappaB and ERK1/2 pathways has been studied [1], but the pathways involved in controlling the switch between its oncogenic and tumor-suppressor functions in different cancers have not been investigated. We discovered IER3 as the most bona fide target of fibroblast growth factor signaling (FGF2) and plays a critical role in the execution of FGF2 dependent pro-survival oncogenic pathways. Our work also implicated the critical role of its antisense RNA partner IER3-AS1 in the oncogenic pathways [16]. Interestingly higher expression correlation between IER3 and IER3-AS1 correlated with their oncogenic functions in cancers resulting in poor clinical outcome. However, in a few cancers like glioblastoma [16] and neuroblastoma (Fig. S2C), we found very poor expression correlation between IER3 and IER3-AS1 transcripts. In these cancers, higher IER3 expression is associated with good prognosis. More importantly, in NB cell lines, IER3 and IER3-AS1 are functionally decoupled through their differential spatial localization. While these two transcripts are preferentially colocalized in the nuclear compartment in HeLa cells, in NB cells, IER3-AS1 is more enriched in the cytoplasm. This distinct spatial segregation suggests that the cellular context plays a key role in modulating the interaction between IER3 and IER3-AS1. Based on these observations, we propose that the differential spatial localization of IER3 and IER3-AS1 in HeLa and NB cells enables IER3 to interact with distinct protein interactomes in a cell type-specific manner. Thus, this compartmentalization could underlie cell type-specific functions, contributing to the differential roles of IER3 in various cancer types.

Our study, for the first time unravels the tumor suppressor properties of IER3 in NB. NB is characterized by great heterogeneity and is the third most common solid tumor and the most common abdominal tumor in childhood and is associated with a poor prognosis and early relapses often cannot be treated successfully. Using p53 wild-type and mutated neuroblastoma cell lines, we demonstrated that IER3 enhances proliferation in the SH-SY5Y neuroblastoma cell line by stimulating DNA replication initiation pathways, which is supported by increased expression of MCM2, MCM4, and RAD51, which are core proteins of the DNA replication initiation complex.

Another interesting aspect of the current investigation is how IER3 differentially regulates its target genes, EGR2 and ADAM19, in a cancer cell-specific manner. Although IER3 binds to both genes in HeLa and NB cell lines, the mechanism by which it drives the differential expression of these two genes in a context-dependent fashion remains unclear. This suggests that additional factors, such as chromatin accessibility and cell type specific transcription factors may contribute to its ability to modulate gene expression in a cell-type specific manner. Our study shows that the differential expression of EGR2 and ADAM19 in HeLa and neuroblastoma cell lines correlates with distinct patterns of H3K4me3 and H3K27me3 chromatin modifications at their promoter regions, indicative of active and repressive chromatin states, respectively. These findings suggest that the regulation of these genes by IER3 is not only dependent on its binding but also cell type specific epigenetic regulators, which may contribute to the cell-type specific expression of these target genes.

EGR2 (Early Growth Response 2), like IER3, is also an immediate early gene that is transiently activated in response to various cellular stimuli, such as growth factors and stress. EGR2 encodes a transcription factor that plays a critical role in regulating downstream target genes involved in processes such as cell growth, differentiation, and apoptosis [27, 31]. Similar to IER3, EGR2 display cell type and context-dependent functions potentially acting as either tumor suppressors or oncogenes in different cancers [27, 32]. Interestingly, the IER3-controlled downstream oncogenic gene regulatory network includes EGR2, FOS, and JUN, all of which are part of the family of immediate early response genes. FOS and JUN are components of the AP-1 transcription factor complex, which plays a critical role in cellular responses to growth signals, stress, and differentiation [33, 34]. Activation of this network, driven by oncogenic mutations, may contribute to early events in tumor progression. Thus, targeting this oncogenic network with small molecules, either alone or in combination with current therapies, presents a promising strategy for treating difficult-to-manage cancers.

Our work demonstrates that ADAM19 play a critical role in IER3 mediated tumor suppression in NB cells. ADAM19 (A Disintegrin And Metalloproteinase 19) belongs to the ADAM family of proteins, is involved in cellular processes such as cell adhesion, migration, and proteolysis of extracellular matrix proteins [35, 36]. While ADAM19 is commonly associated with development and tissue homeostasis [35], studies have shown that it can be upregulated in certain cancers and exert tumor-suppressive effects. For example, in prostate cancer ADAM19 expression has been correlated with better prognosis and decreased metastasis, indicating its potential role in suppressing invasion and metastasis [37]. Similarly, in other cancer types such as ovarian cancer, ADAM19 has been shown to inhibit cell migration and invasion [38].

**Fig. 7:**
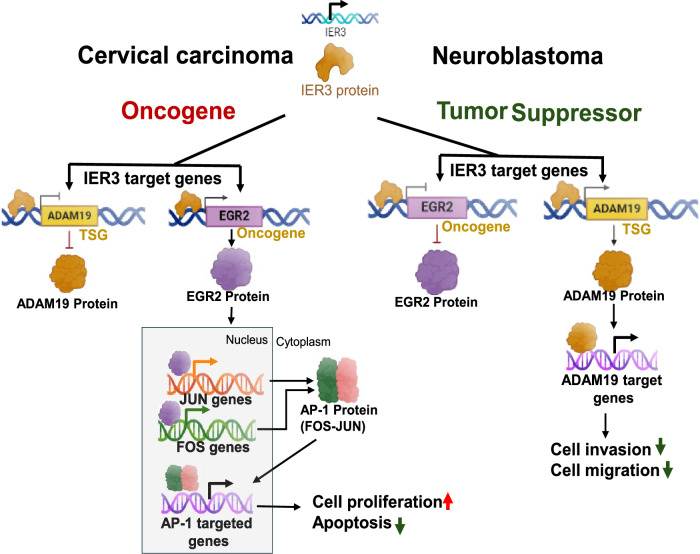
Overall summary of IER3 oncogenic and tumor suppressor functions. Schematic illustration of oncogenic and tumor suppressor functions of IER3 and its target genes in cervical carcinoma and neuroblastoma cell lines.

Specifically, we show that EGR2 promotes oncogenic activity through the c-JUN and c-FOS signaling pathway, which are associated with cellular proliferation and survival in cervical carcinoma. In contrast, ADAM19 functions as a tumor suppressor by modulating cell invasion and migration pathways, contributing to the inhibition of metastatic potential in neuroblastoma (Fig. 7). Hence, for the first time, we identify EGR2 and ADAM19 as critical regulators of these IER3-mediated functions, playing distinct roles in the progression of these cancers.

Overall, our findings highlight that IER3 overexpression could enhance therapeutic outcomes in neuroblastoma by suppressing tumor growth. In contrast, targeting IER3 in cervical carcinoma could reduce oncogenic signaling and tumor progression. Therefore, therapeutic strategies aimed at modulating IER3 expression, depending on its role in specific cancers, may provide novel, targeted treatment options for these distinct malignancies.

## Methods

### Cell line maintenance, transient and stable transfections

HeLa, SH-SY5Y, SK-N-BE(2) and KELLY cells were obtained from CLS Cell Lines Service. All the cell lines except SK-N-BE(2) were cultured using DMEM supplemented with 10%FBS and 1X penicillin and streptomycin. For culturing SK-N-BE(2), DMEM-F12 media was used with supplements as mentioned above. siRNAs were obtained from Sigma Aldrich and transfected using Lipofectamine RNAiMAX reagent using manufacturer instructions. The control siRNA, siRNA sequences/siRNA ids targeting different genes are described in the Supplementary Table. Transfection of the plasmid DNA was performed using Lipofectamine 2000 reagents using manufacturer instructions. PCDNA3.1 vector is used as control for DNA transfections. Stable cell lines with KD and OE cells were performed using Lentiviral shRNA particles targeting IER3 and non-target shRNA control which were procured from Sigma Aldrich. Stable shRNA cells were generated using the method as described previously [39]. The sequence of the shRNAs used for each RNAs and the expression vectors used for over-expression of TFPI, ADAM19, and EGR2 have been provided in the Supplementary Table. All the over expression vectors were bought from SinoBiological, Germany and the expression levels of down regulation and overexpression of the target genes was verified using RT-qPCR and western blotting.

### RNA isolation and RT-qPCR

Total RNA was isolated from cell lines using ReliaPrep RNA cell miniprep system (Promega). All RNA fractions were resuspended in TE (pH 7) and quantified using a Nanodrop-1000 spectrophotometer (Nanodrop Technologies) and tested for DNA contamination by real-time RT-PCR lacking reverse transcriptase. cDNA synthesis is performed using Improm-II Reverse Transcriptase kit (Promega) and RT-qPCR analysis was done using Power SYBR Green PCR master mix (Applied Biosystems). Differences in expression were calculated using the ΔΔCt method. Error bars represent the SD of at least three independent experiments and p-value derived from a two-sided unpaired Student’s t-test. All the primer sequences used for RT-qPCR were mentioned in Supplementary Table.

### Immunobloting

For protein extraction, Cells were washed in 1X cold PBS and lysed in RIPA lysis buffer (Sigma Aldrich; 20-188) and complete protease inhibitor cocktail. The cell lysates were spun down at maximum rpm for 20 min at 4 °C and the supernatants estimated for protein concentration using Pierce BCA Protein Assay Kit (Thermo Scientific; 23225) according to the manufacturer’s instructions. 30 μg of total cell lysates were resolved by SDS PAGE on NuPAGE Novex 4–12% Bis-Tris Protein Gels (Invitrogen). Following the electrophoresis, proteins were transferred on to 0.45 μm nitrocellulose membrane (Hybond ECL, Amersham, GE healthcare). Membranes were blocked in 5% BSA in 1X TBS-T (10 mM Tris-base pH 7.5, 150 mM NaCl, and 0.1% Tween) for 1 h at room temperature followed by overnight incubation with primary antibody prepared in fresh blocking solution. Membranes were washed thrice for 5 min each in 1X TBS-T followed by incubation with anti-mouse or rabbit secondary antibodies for 1 h at room temperature in 5% skim milk made in 1X TBS-T. Membrane washes were repeated 3 times in 1XTBS-T as above followed by visualizing protein bands with chemiluminescent Substrate (Thermo Scientific) using IMAGE BioRad 4.0 alpha software. The list of all the antibodies used for this study are mentioned in Supplementary Table.

### Cell migration/invasion and wound healing assays

The migration and invasion assay was performed using the BioCoat Matrigel Invasion Chamber (Thermofischer scientific, 11553570) as per the manufacturer’s instructions. In brief, cells were seeded approximately 50,000–60,000 cells per ml in density on the upper chamber of the biocoat invasion chamber in Dulbecco’s modified Eagle’s starvation medium without serum. Medium with 10% serum was added to the lower chamber. After 48 h, the migrated cells were fixed and stained with DAP1 (4’,6-diamidino-2-phenylindole) and images were taken in EVOS FL Auto Imaging System (Thermo Fischer scientific) and colonies were counted using imageJ software. Wound healing assay was performed using a previously described protocol by Chun-Chi Liang et al. [40]. Cell migration was determined by the rate of cells moving towards the scratched area. All experiments were plated in triplicate wells and were carried out at least three times.

### Cell proliferation assay

We carried out the proliferation assay using CellTiter-Glo™ luminescent cell viability assay kit (Promega, USA) according to the manufacturer’s instructions. In the case of transient or inducible KD, we assayed the proliferative cells 96 h post-treatment, respectively. In stable KD cells, we assayed the viability 48 h after seeding an equal number of cells. Statistical analyses were performed using 3 independent biological replicates to calculate the p-value derived from a two-sided unpaired Student’s t-test.

### Cell colonization, cell cycle analysis and apoptosis assay

We assessed the clonogenicity of the tested cells using standard 6-well plates. We seeded individually suspended 5000 cells/well of each cell line and allowed them to grow for one week. The proliferating cells were washed with PBS, fixed with 100% methanol for 20 min at room temperature and then stained with 0.5% crystal violet in 25% methanol. Excess stain was washed away several times with dH2O, and the plates were left to dry. Stained colonies were photographed using a digital camera.

The Cell cycle profiles of different cell lines and percentage of Apoptotic cells were analysed using the NucleoCounter NC-3000 platform (Chemometec, Denmark). We checked the profiles of stable KD cells after 48 h post-seeding. The cells were collected, washed with PBS, and fixed in 70% ethanol at −20 °C overnight. We stained the fixed cells using DAPI solution (Chemometec, Denmark) for 10 min at 37 °C and analyzed the cell cycle profiles according to the manufacturer’s instructions. The data was analyzed from Nucleocounter cell cycle analysis software and the graphs were plotted with those values using GraphPad Prism7.

Apoptotic cells were measured using Annexin V Assay kit (Chemometec, Denmark) according to the manufacturer’s instructions. Histograms show the Annexin V-CF488A intensity of the cell population. Scatter plots show the Annexin V-CF488A intensity versus the intensity of Propidium iodide (PI).

### RNA-sequencing, differential expression and pathway analysis

RNA from both *IER3sh and* CRISPR/cas9 promoter deletion knockdown samples of with two independent shRNAs were sequenced in duplicate sets using Illumina Sequencing Platform. The Illumina sequenced reads were aligned to hg38 (GRCh38) version of the genome using Hisat2 aligner and quantified using featureCounts (Subread-1.4.5). The differential expression of treated and untreated samples was performed using DESeq using R package. Adjusted p-value of 0.05 by Benjamini-Hochberg method and log2FC of 1 is considered as significant. Functional enriched biological pathways of differentially expressed protein coding genes were determined using GeneSCF (FDR < = 0.05). The differentially expressed genes were considered significant with adjusted *p* value of 0.05 by Benjamini-Hochberg.

### ChIP sequencing and analysis

ChIP was performed with the Ideal ChIP seq kit (Diagenode, C01010051) according to the manufacturer’s protocol using an IER3 polyclonal antibody (Invitrogen, PA5-20391). ChIP DNA was quantified using Qubit 2 Fluorometer (ThermoFischer Scientific, Q32866) and around 10 ng of ChIP DNA was used to prepare sequencing libraries using the ThruPLEXFD Prep Kit (Rubicon Genomics, Ann Arbor, USA) according to the manufacturer’s specifications. The final DNA libraries were sequenced (PE50) using an Illumina HiSeq 2500 instrument with ~40 to 50 million reads per sample.Reads were filtered based on quality with TrimGalore (0.4.0) (https://github.com/ FelixKrueger/TrimGalore). Alignment to hg19 was performed with Bowtie2 (2.2.6) with default parameters. Quality measures such as cross-correlation, cumulative enrichment and clustering were performed using phantompeakqualtools (1.1) (http://code.google.com/p/phantompeakqual-tools/) and deepTools (2.0.1). Duplicates are labeled using SAMBLASTER (Faust et al., 2014) and mapping quality value is calculated by MAPQ. Peak calling was carried out with MACS2 (2.1.0.20140616) (https://pypi.python.org/pypi/ MACS2) with a minimum FDR (q-value) cutoff for peak detection of 0.05. Peaks were normalized with their corresponding input using deepTools2. The weighted score (W-score) was calculated by dividing the average peak score (AveS) by range of the peak (R) for geneG and multiplying by the number of peaks associated with the gene G (N); (AveS/R*N)*100.

For validation ChIP RT-qPCR was performed using Power SYBR Green PCR master mix (Applied Biosystems, Warrington, UK) for the presence of the IER3 target genes, Primers were designed at TSS, upstream and downstream to TSS. All the primer sequences are listed in Supplementary Table. The normalized percentage of input and fold enrichment values for ChIP data were calculated with the ΔΔCt method from the Excel-based ChIP-qPCR analysis template.

### Mouse xenograft tumor model

We performed all animal experiments according to the ethical permit (No: 5.8.18-02708/2017), reviewed and approved by the Animal Ethical Review Board, University of Gothenburg, Sweden. 10 × 10^6^ stable either control (Ctrl)Sh or IER3sh RNA knockdown SH-SY5Y and SK-N-BE(2) cells were subcutaneously injected on the dorsal back region of 5- to 6-week-old NSG™ mice (Charles River, France) (*n* = 6) with 15% Matrigel (Corning). Following 2–3 weeks post-engraftment, we measured the tumors volumes. Formalin-fixed mice xenografts were embedded in paraffin and Immunohistochemical analyses were performed on sections of 2–4 μm using an antibody against Ki-67 (proliferation marker). Ki-67 images were taken using EVOS FL Auto Imaging System (Thermo Fischer scientific).

### Statistical analysis

The data are expressed as the means ± standard deviations (SD). The statistical significance of differences between two groups was analysed by two-tailed unpaired Student’s *t* test. Comparisons among three or more groups were performed by two-way analysis of variance (ANOVA) with the Dunnett’s multiple comparison test for multiple comparisons. Differences for which *p* < 0.05 were considered statistically significant.

## Supplementary information

Supplemental File

## Data Availability

GEO accession numbers for RNA-seq and ChIP-seq data are GSE279085 and GSE279084. The data will be released to public upon acceptance of the paper and the token numbers for visualizing the data until then are mentioned below; RNA-seq https://www.ncbi.nlm.nih.gov/geo/query/acc.cgi?acc=GSE279085 (Reviewer Access Token: qzspeaawhbwjpmb) Chip-seq: https://www.ncbi.nlm.nih.gov/geo/query/acc.cgi?acc=GSE279084 (Reviewer Access Token: kpgzokukvfqllwp).
