# Supplementary material for: IER3: exploring its dual function as an oncogene and tumor suppressor
Source: Cancer Gene Ther. 2025 Mar 16;32(4):450–63. doi: 10.1038/s41417-025-00891-y (PMC11976266; doi:10.1038/s41417-025-00891-y)
Supplement: Supplementary file 1 — Supplemental File [file 41417_2025_891_MOESM1_ESM.pdf]

FIGURE S 1

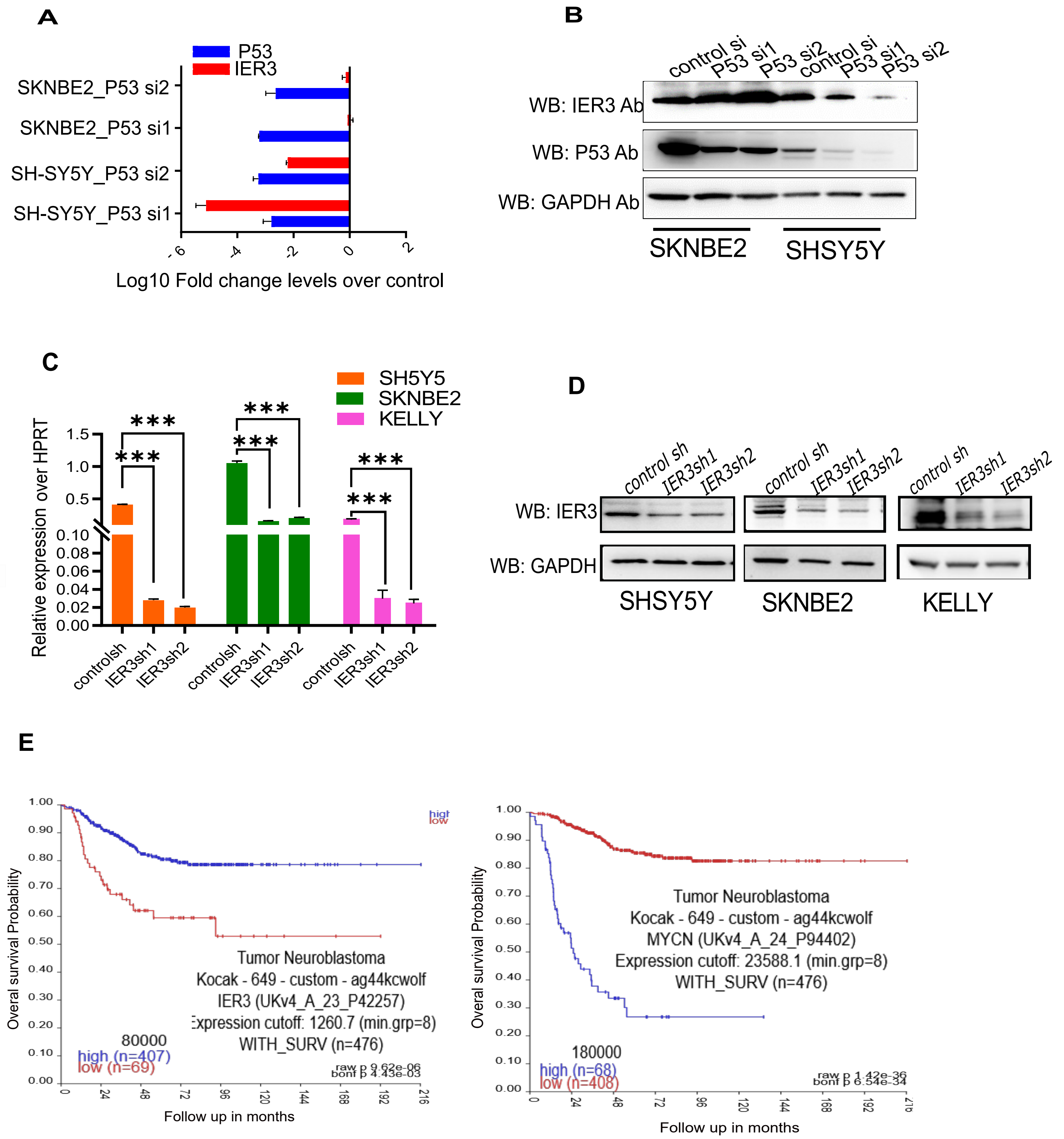

Figure S1: A) RT-qPCR expression levels of IER3 and P53 genes upon P53 siRNA mediated down-regulation in SH-SY5Y and SK-N-BE(2) NB cell lines. B& D) Western blot showing the protein levels of IER3 and P53 in NB cell lines. GAPDH protein levels is used as internal control. C) RT-qPCR analysis showing mRNA and protein expression levels of sh mediated IER3 downregulated SH-SY5Y, SK-N-BE(2) and KELLY NB stable cell lines. E) Survival plots for IER3 and MYCN expression in the same cohort showing inverse correlation of expression vs survival probability.

FIGURE S2

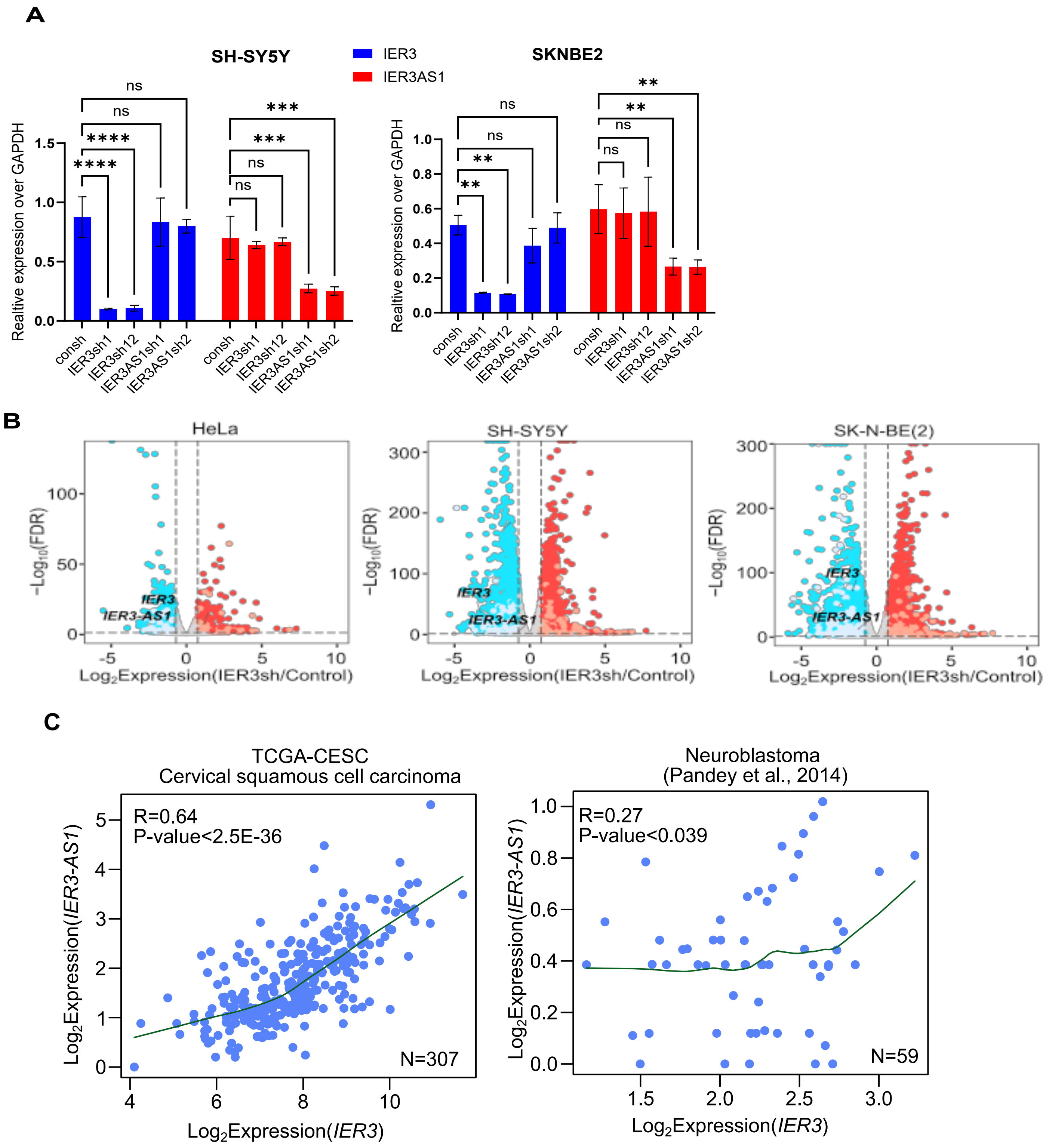

Figure S2: A) RT-qPCR plots showing expression levels of IER3 and IER3AS1 in IER3sh NB cell lines. B) Volcano plots showing Log10FDR on Y axis and LogFC on X-axis of DEGs from RNA-seq data of HeLa and NB cell lines. Up and down regulated DEGs are shown in red and blue color dots respectively. The non-significant DEGs are represented as gray dots. FDR threshold (=0.05) is reported as horizontal dotted gray line. FDR values are derived from DESeq2 R-package by adjusting p -values using Benjamini -Hochbergmethod. C) Expression correlation plots of IER3 and IER3 -AS1 in cervix squamous carcinoma and Neuroblastoma cancers from TCGA and published databases . Correlation co -efficient and p -value are mentioned above for each plot.

FIGURE S3

A

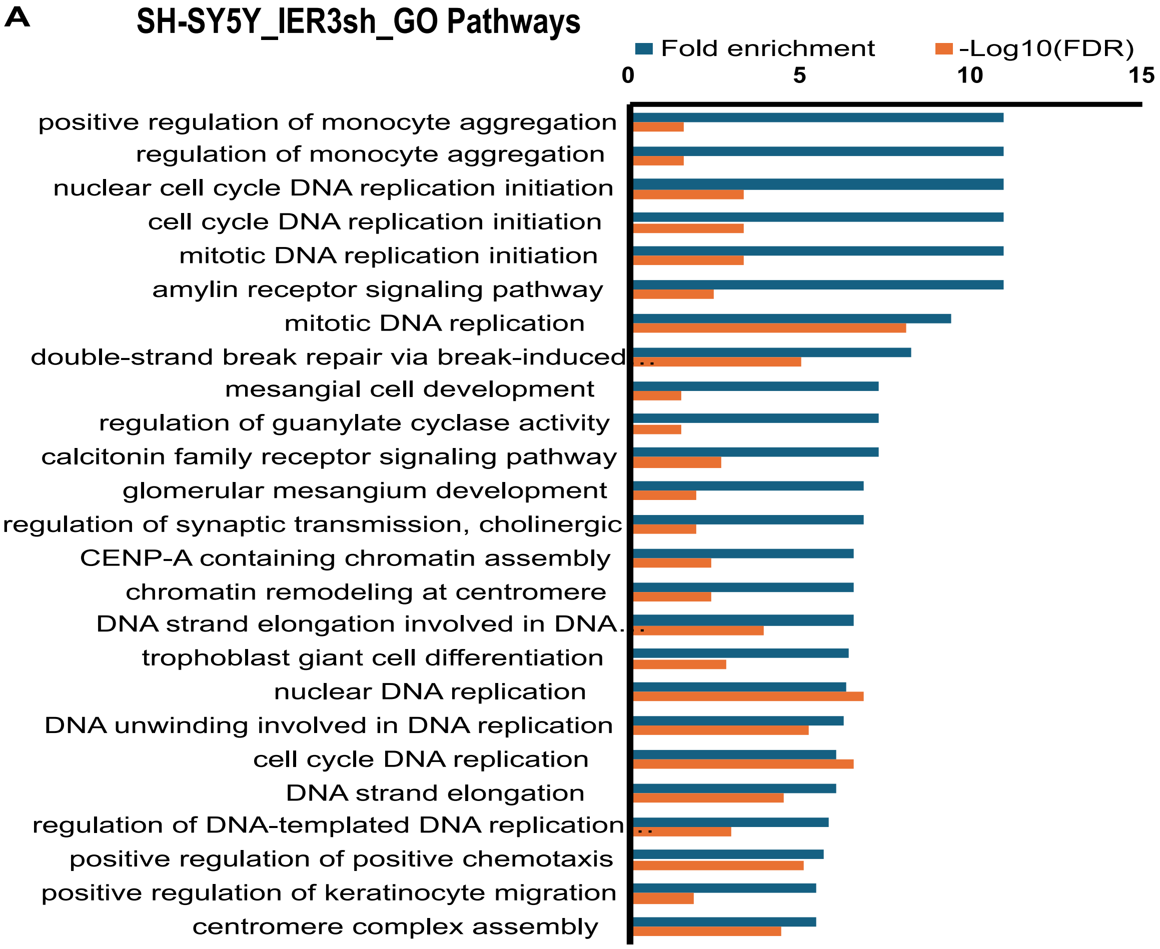

B

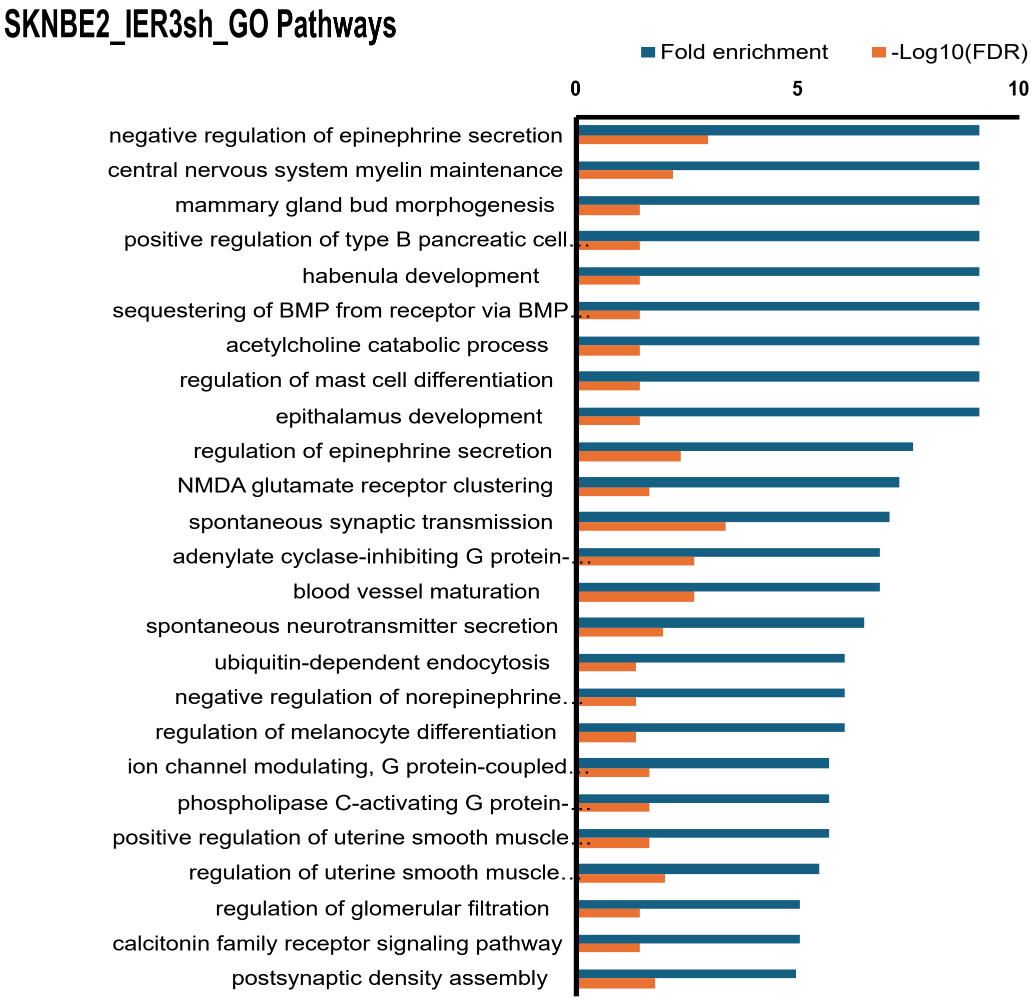

Figure S3: A -B) Functional gene enrichment GEO Pathway analysis for differentially expressed consh Vs IER3sh samples from RNA sequencing data of SH -SY5Y and SKNBE2 cell lines respectively. C) Pie diagram showing the percentages of the top pathways from GEO pathway analysis in SH-SY5Y and SKNBE2 cell lines.

FIGURE S4

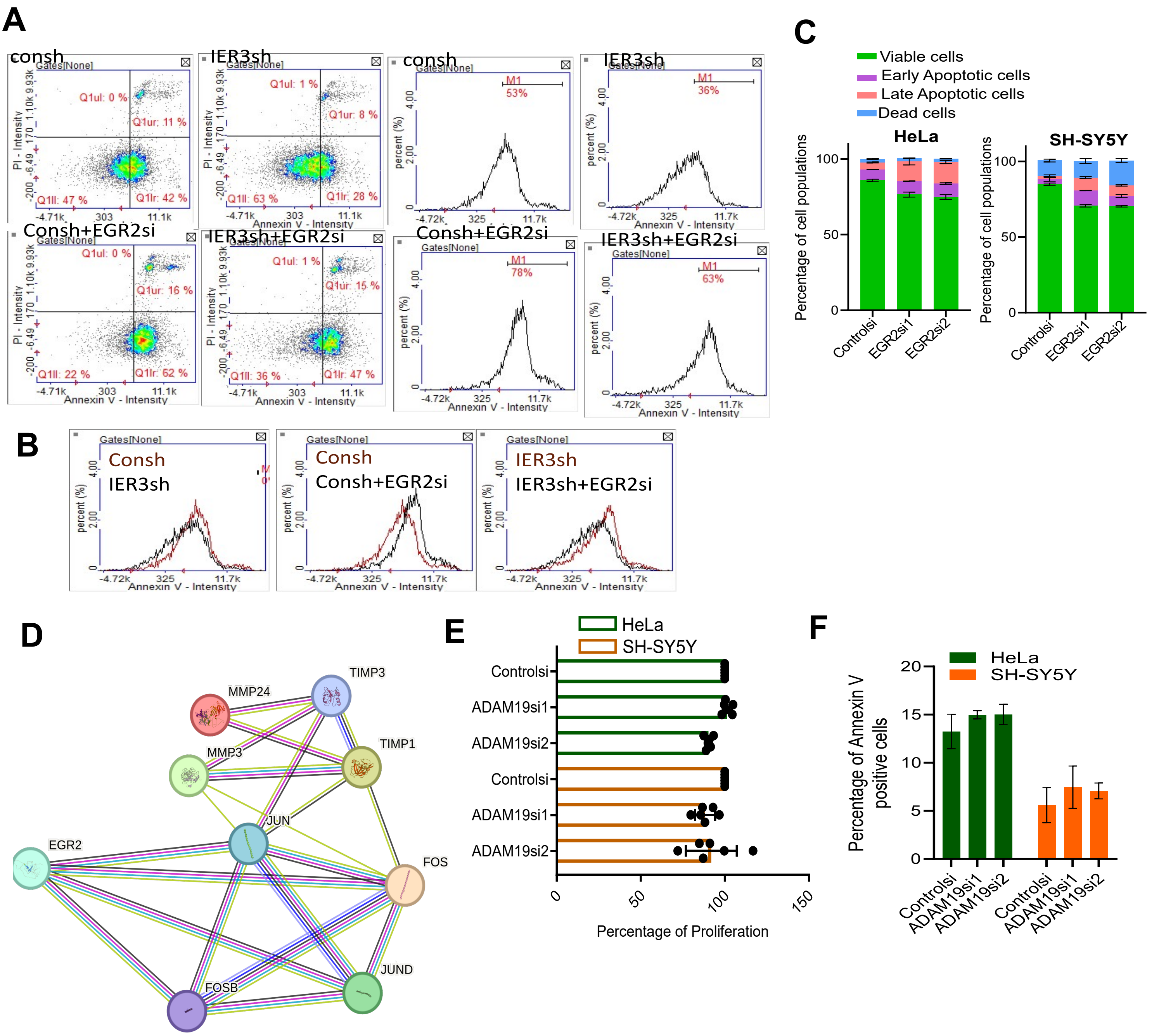

Figure S4: A) Scatter Plots (Left side) and Histograms (Right side) showing Annexin V positive cells (Apoptotic cells) EGR2 siRNA KO in SH -SY5Y consh and IER3sh stable cell lines. B) The histograms showing the overlap peaks of both consh/IER3sh; consh/consh with EGR2si and IER3sh/IER2sh with EGR2 siRNA samples. Cells were stained with Annexin V - Fluorescein Isothiocyanate (FITC) conjugated antibody and propidium iodide (PI) and analyzed using NucleoCounter NC-3000. Quadrants (ll, ul, ur and lr) and marker (M2) in the displayed plots were used to demarcate various cell populations. In the right two quadrants, ur and lr show the percentage of Annexin V and PI positive cells. C) Bar plots showing percentage of cell populations for viable, early apoptosis, late apoptosis and dead cells for consi and EGR2si KO samples in HeLa and SH-SY5Y5 cells. D) Figure showing STRING analysis on C-JUN/FOS pathway genes. E) and F) Cell viability assay and Annexin V assay showing percentage of proliferation cells i and Annexin V positive cells respectively in consi and ADAM19 si KO samples of HeLa and SH-SY5Y cells.

LIST OF RT-PCR PRIMERS USED IN THIS STUDY

Gene	Primer sequences (5'-3')
IER3_RTPCR_FP	TCTTCACCTTCGACCCTCTC
IER3_RTPCR_RP	ACACCCTCTTCAGCCATCAG
IER3-AS1_RTPCR_FP	GGATGCTGGGTTCTGTGACT
IER3-AS1_RTPCR_RP	GCGGACCATTAGGAATGAGA
HPRT_RTPCR_FP	GCTATAAATTCTTTGCTGACCTG
HPRT_RTPCR_RP	AATTACTTTTATGTCCCCTGTTGACTG G
P53_RTPCR_FP	CCCAAGCAATGGATGATTTGA
P53_RTPCR_RP	GGCATTCTGGGAGCTTCATCT
BRCA2-RTPCR_FP	TCCAAAGAGAGGCCAACATT
BRCA2-RTPCR_RP	TCAGCCCTTGCTCTTTGAAT
CDC45-RTPCR_FP	ACCTTGAAGTTCCCGCCTAT
CDC45-RTPCR_RP	CTGCTCGTAGTCAAAGAGGATG
GINS1-RTPCR_FP	CCTTCAACGAGGATGGACTC
GINS1-RTPCR_RP	TACAGTGCAGCGTCGATTTC
GINS3-RTPCR_FP	CGCCATGTCAGAGGCTTATT
GINS3-RTPCR_RP	AGTCCTCCAACCCTCTTGGT
LIG1-RTPCR_FP	CTGGCGGATCTGAGTGTGT
LIG1-RTPCR_RP	GCCTCCTTCTCAGGCTTCTT
MCM2-RTPCR_FP	ATTGGAGATGGCATGGAAAG
MCM2-RTPCR_RP	GTCCTCCTCATCGCTGTCAT
MCM3-RTPCR_FP	CGCAGTGTCCACTACTGTCC
MCM3-RTPCR_RP	CTCCTGGATGGTGATGGTCT
MCM4-RTPCR_FP	TGTTATTGGTGAGCCATTTTT
MCM4-RTPCR_RP	TGAGTCAGGGTAACGGTCAA
MCM6-RTPCR_FP	AATCCTCGGCACTAAGCAAA
MCM6-RTPCR_RP	GGACGAATCAGTTCCTCTGC
POLA1-RTPCR_FP	GGCGAGTGCTCTGTCAGATT
POLA1-RTPCR_RP	GCCTGAACCAGCTTCGAATA
RAD51-RTPCR_FP	AGACCGAGCCCTAAGGAGAG
RAD51-RTPCR_RP	TCTGCATTTGCTTCAAGCTG
CDH5-RTPCR_FP	CCAACGGAACAGAAACATCC
CDH5-RTPCR_RP	CAATGTGCATCTGGTTCCAA
MMP2-RTPCR_FP	CTACGATGGAGGCGCTAATG
MMP2-RTPCR_RP	TGTCCTTCAGCACAAACAGG
RECK-RTPCR_FP	ACCAAATGTGCCGTGATGTA
RECK-RTPCR_RP	TCCAAGGCAATAGCCAGTTC
ACVRL1-RTPCR_FP	AGGAAAGGCCTTCTGATGCT

ACVRL1-RTPCR_RP	AATGTGGGCTCTCACACG
S1PR1-RTPCR_FP	CAGCAGCAAGATGCGAAG
S1PR1-RTPCR_RP	AGAGCCTTCACTGGCTTCAG
EGR2_RTPCRFP	TTGACCAGATGAACGGAGTG
EGR2_RTPCRRP	GTTGAAGCTGGGGAAGTGAC
ADAM19_RTPCRFP	CTCTGCTTGCTGGCGTTT
ADAM19_RTPCRRP	CTTTTCTCTCACGGGGCTTT
FOS_RTPCR_FP	ATGATGTTCTCGGGCTTCAA
FOS_RTPCR_RP	GTGGGAATGAAGTTGGCACT
FOSB_RTPCR_FP	TCTGTCTTCGGTGGACTCCT
FOSB_RTPCR_RP	CCTGGTGTGGAGTAGCTGGT
JUN_RTPCR_FP	CCCCAAGATCCTGAAACAGA
JUN_RTPCR_RP	CCGTTGCTGGACTGGATTAT
JUND_RTPCR_FP	GCCCTGGAGGATTTACACAA
JUND_RTPCR_RP	CTCAGGTTCGCGTAGACAGG
MMP24_RTPCR_FP	CGGGCAGAACTGGTTAAAGT
MMP24_RTPCR_RP	TCGATCGTTGTCTGATCCAA
NEAT1_RTPCR_FP	CAGTTAGTTTATCAGTTCTCCCATCCA
NEAT1_RTPCR_RP	GTTGTTGTCGTCACCTTTCAACTCT
TIMP3_RTPCR_FP	CCTTCTGCAACTCCGACATC
TIMP3_RTPCR_RP	CCACAGAGACTCTCGGAAGC
TIMP1_RTPCR_FP	GCTTCTGGCATCCTGTTGTT
TIMP1_RTPCR_RP	CGCTGGTATAAGGTGGTCTG

**LIST OF ChIP PRIMERS USED IN THIS STUDY**	
Gene	Primer sequences (5'-3')
EGR2_TSS_ChIPFP	CCCCTCGCCGAGCTATTA
EGR2_TSS_ChIPRP	CAGCGACGTCACGGGTAT
EGR2_UP_ChIPFP	GCTTTCCAAGGACTCACACC
EGR2_UP_ChIPRP	CAGCGGAGGTTCAATAAAGG
EGR2_DS_ChIPFP	CACTCCGTTCATCTGGTCAA
EGR2_DS_ChIPRP	AGTTGGGTCTCCAGGTTGTG
ADAM19_TSS_ChIPFP	CGGGCATTTTATGTCCTACC
ADAM19_TSS_ChIPRP	CTTCTCGCCTTCTCCTTCCT
ADAM19_DS_ChIPFP	GGAGGGAGTGATGAAGACCA
ADAM19_DS_ChIPRP	CCCTAACCATCCCCAATTCT
ADAM19_UP_ChIPFP	AGAACCTCCTCTGTGCCTCA
ADAM19_UP_ChIPRP	GGACCAACAGTCAGCGTTTT

**LIST OF siRNAs USED IN THIS STUDY**

Name	siRNA ID	Company
P53 siRNA1	SASI_Hs02_00302767	Sigma Aldrich
P53 siRNA2	SASI_Hs02_00302768	Sigma Aldrich
EGR2 siRNA1	SASI_Hs01_00170423	Sigma Aldrich
EGR2 siRNA2	SASI_Hs02_00302474	Sigma Aldrich
ADAM19 siRNA1	SASI_Hs01_00151472	Sigma Aldrich
ADAM19 siRNA2	SASI_Hs01_00151475	Sigma Aldrich
MCM2 siRNA1	SASI_Hs01_00013911	Sigma Aldrich
MCM4 siRNA1	SASI_Hs01_00218872	Sigma Aldrich
RAD51 siRNA1	SASI_Hs01_00018925	Sigma Aldrich
Mission siRNA negative control	S1C001	Sigma Aldrich

**shRNAs target sequence**		
**Name**	**Target Sequence**	**Company**
IER3_Oligo2_S	GGGCTCCGAAGTCAGATTAAA	Sigma Aldrich
IER3_Oligo2_AS	GGGCTCCGAAGTCAGATTAAA	Sigma Aldrich
IER3_Oligo3_S	GGTACGCCTGGTGTTTCTTTG	Sigma Aldrich
IER3_Oligo3_AS	GGTACGCCTGGTGTTTCTTTG	Sigma Aldrich
IER3AS1_Oligo2_S	TGATTGCCACATCTCGGATTC	Sigma Aldrich
IER3AS1_Oligo2_AS	TGATTGCCACATCTCGGATTC	Sigma Aldrich
IER3AS1_Oligo3_S	ACCGCAGACTGGGCAATGAAA	Sigma Aldrich
IER3AS1_Oligo3_AS	ACCGCAGACTGGGCAATGAAA	Sigma Aldrich
Control shRNA particle from Sigma Aldrich		

**sgRNAs for IER3 promoter KO CRISPR clone**		
**Name**	**Target Sequence**	**Company**
**IER3Prom_UPsgRNA1_forw**	**TCGGCGATACTCACCACTCG**	Sigma Aldrich
**IER3Prom_UPsgRNA1_Rev**	**CGAGTGGTGAGTATCGCCGA**	Sigma Aldrich
**IER3Prom_DSsgRNA1_forw**	**TAAAGGTGCAAAGACTGTAT**	Sigma Aldrich
**IER3Prom_DSsgRNA1_Rev**	**ATACAGTCTTTGCACCTTTA**	Sigma Aldrich

**Overexpression vectors**		
**Name**	**Target Sequence**	**Company**

EGR2 cDNA ORF Clone, Human, untagged	Cataloge number- HG14868-UT	Sino Biological
ADAM19 cDNA ORF Clone, Human, C-DYKDDDDK (Flag®) tag	Cataloge number- HG18760-CF	Sino Biological
**Plasmid contructs**		
**Product**	**Company**	**Identifier**
lentivirus Packaging contructs		
psPAX2	Addgene	#12260
PMD2G	Addgene	#12259

**<u>Western blot antibodies</u>**		
Antibodies	**Company**	**Identifier**
IER3	Abcam	ab65152
GAPDH	Santa Cruz Biotech	sc-25778
P53	Proteintech	10442-1-AP
MCM2	Proteintech	10513-1-AP
MCM4	Proteintech	13043-1-AP
RAD51	Proteintech	14961-1-AP
Phospho-Histone H2A.X (Ser139) Polyclonal antibody	Proteintech	29380-1-AP
PARP	cell signaling	9532S
Caspase3	Abcam	ab32042
EGR2		13491-1-AP
ADAM19	Thermofischer	PA5-26816
JUN	Proteintech	24909-1-AP
C-FOS	Proteintech	66590-1-Ig
**<u>ChIP antibodies</u>**		
Antibodies	**Company**	**Identifier**
IER3	Thermofischer	PA5-20391
H3K27me3	Diagenode	C15410195
H3K4me3	Diagenode	C15410003-50
**<u>Immunostaining antibodies</u>**		
Antibodies	**Company**	**Identifier**
Ki67	Abcam	Ab16667

Phospho-Histone H2A.X (Ser139) Polyclonal antibody	Proteintech	29380-1-AP
**Nucleocounter staining antibodies**		
Antibodies	**Company**	**Identifier**
Annexin V	Thermofischer Scientific	A13201
PI	Chemometec	910-3016

Experimental Models

**Product**	**Company**	**Identifier**
HeLa cell line	ATTC	HeLa (CCL-2)
SH-SY5Y cell line	ATTC	SH-SY5(CRL-2266)
SK-N-BE(2)	ATTC	SK-N-BE(2)CRL-2271

Chemicals, Kits, Enzymes and other reagents

**Product**	**Company**	**Identifier**
CellTiter-Glo 3D Cell Viability assay kit	PROMEGA	G9683
Crystal Violet	Sigma Aldrich	HT90132
ProLong™ Gold Antifade Mountant with DAPI	Thermo Scientific	P36935
Power SYBR™ Green PCR Master Mix	Thermo Fisher	4368708
iDeal ChIP-seq kit for Histones	Diagenode	C01010051
SuperSignal West Pico PLUS Chemiluminescent Substrate	Thermofisher	34580
Pierce BCA Protein Assay Kit	Thermo Scientific	23227
Ultra Pure BSA	Thermo Scientific	10743447
Tween-20	Sigma Aldrich	11332465001
FBS	Gibco	A5256701
DMEM, high glucose, GlutaMAX™ Supplement, pyruvate (10x500ml)	Thermo Fisher	31966047
DMEM/F-12, GlutaMAX™ Supplement (10x500ml)	Thermo Fisher	31331093
Penicillin-Streptomycin	Gibco	15140-122

Opti-MEM™ Reduced Serum Medium	Thermofisher	31985062
CalPhos™ Mammalian Transfection Kit	Takara	631312
Lipofectamine RNAiMAX Transfection Reagent	Thermo Scientific	13778150
Lipofectamine 2000 Reagent	Thermo Scientific	11668-019
